# Editorial: Current questions and challenges in healthcare of the post-socialist countries

**DOI:** 10.3389/fpubh.2023.1254898

**Published:** 2023-08-15

**Authors:** Yuriy Timofeyev, Maria Kaneva, Mihajlo Jakovljevic

**Affiliations:** ^1^Department of Strategic and International Management, HSE University, Moscow, Russia; ^2^Institute of Economics and Industrial Engineering, Siberian Branch of the Russian Academy of Sciences, Novosibirsk, Russia; ^3^Institute of Advanced Manufacturing Technologies, Peter the Great St. Petersburg Polytechnic University, Saint Petersburg, Russia; ^4^Institute of Comparative Economic Studies, Hosei University, Tokyo, Japan; ^5^Department of Global Health Economics and Policy, University of Kragujevac, Kragujevac, Serbia

**Keywords:** health care system's, health expenditure, health insurance, health literacy, COVID-19 vaccine hesitancy, post-socialist countries

In our Research Topic, the contributing authors discuss diverse issues related to the healthcare reforms in the post-Soviet bloc: Bulgaria, China, Croatia, Kazakhstan, Poland, Russia, and Syria. In all countries, healthcare has been an important determinant of the GDP growth in the last decades (1).

In 1991, Russia introduced mandatory health insurance (MHI) in place of the Semashko system. Shishkin and Sheiman assess the reform progress. The MHI model has contributed to more sustainable health funding and pooling through geographical equalization. However, the implementation of the purchasing function still has many problems. There is no accounting for the interventions' quality of care or cost-effectiveness. The current purchasing care approach hinders the development of new medical technologies. Among the challenges of the MHI regime is that it is not separated from the state budgetary system, and the state regulates its actual performance. The actors of MHI do not have sufficient motivation to improve the health care system's performance. Developing the competitive MHI model requires long-term efforts from health policymakers. Russia's health reform challenges are widely shared among the BRICS health systems (2).

Reforms brought other changes to the healthcare sector. Sheiman reports that the traditional hospital-centered service delivery model in Russia results in a shortage of doctors in outpatient care, for example, cardiologists [see (3)], and a surplus in hospital care. This surplus increased by 21% in 2016–2019. Another reason for the labor shortage in outpatient care is the lack of medium- and long-term labor planning by regional governments and medical universities. The author recommends policymakers to assess the needs of medical personnel in a region and then make commitments for subsidies for the education of students of the demanded professions and the employment of future graduates. The quotas for post-graduate training should be developed not 1.5 years before the start of admission (as it is now), but 3–4 years before. It is also advised to shift the focus from inpatient care to outpatient care, including primary care. District physicians should be retrained as general practitioners (GPs). Such a measure will reduce the current shortage of medical specialists in outpatient care since a GP can partly fulfill the role of a specialist.

Another common challenge in the current healthcare of post-Soviet countries is little or no access to up-to-date international literature or opportunities for continuing medical education (4). Walkowiak et al. study the awareness of rare diseases (RDs) among medical students and practicing physicians in Kazakhstan. The authors surveyed 308 individuals at the Aktobe Medical University to assess the level of knowledge of RDs and their prevalence in Kazakhstan. The results demonstrate that students and medical doctors lack knowledge about the etiology, epidemiology, and prevalence of rare diseases in the country and are unaware of the existence of the central register of RD patients and reimbursement schemes for orphan drugs. The authors recommend moving away from the Soviet teacher-centered course-based approach and instead promoting student-centered methods of instruction and elective courses, including on RDs and orphan drugs. These measures will allow faster diagnosis of RDs and their treatment in Kazakhstan.

Franic presents the topic of COVID-19 vaccine hesitancy and explores the political and economic factors which shape the attitudes to vaccination in transition economies. The paper employs the data from Flash Eurobarometer conducted in May 2021 in EU countries. Transition economies recorded lower levels of immunization than developed countries. Using multinomial regression models, the author shows that the viewpoint on vaccination is shaped by distrust in the authorities and government related to the socialist legacy. Besides general trust in government, satisfaction with democratic principles in society, trust in science, and specific views on how the authorities handled the pandemic are the critical determinants of attitudes toward vaccination against COVID-19 in the EU. Transition countries require wide-ranging reform to restore citizens' trust in government.

The COVID-19 pandemic has altered how we interact with society from offline to online communication [see, e.g., (5, 6)]. Therefore, the concept of health literacy, the degree to which individuals can find, process, and use information to inform health-related decisions and actions, has become very relevant. Kaloyanova et al. assess the health literacy of university students from the faculty of mathematics and informatics at Sofia University (Bulgaria) based on a COVID-HL survey. The results show that although computing students are skilled at searching for, allocating, and evaluating health information, they do not feel confident about the future. Furthermore, they do not have a clear view and knowledge of what to do with the health information they have. The study demonstrates that important decisions relating to health should be taken by an individual together with a health professional.

Global environmental pollution is considered an international public health issue. China has made significant progress in improving its healthcare system and reducing pollution (7). Wu et al. discuss the impact of environmental pollution liability insurance (EPLI) on the corporate environmental performance of Chinese enterprises in heavily polluting industries. EPLI is a type of insurance that compensates for injuries and deaths caused by pollution. The study, employing fixed-effects regression analysis, shows that EPLI has a positive impact on corporate environmental performance. First, EPLI increases the pressure of stakeholders' environmental demands on company managers, thus prompting managers to adopt green measures to improve the company's environmental performance. Second, EPLI is more effective in improving the environmental performance of companies with higher public visibility. Finally, compared with state-owned enterprises, EPLI has a more significant positive effect on the corporate environmental performance of non-state-owned enterprises.

Very high growth rates of healthcare expenditure remain a hot issue in Central and Eastern Europe. Considering the case of Bulgaria, Mitkova et al. analyse the impact of budget capping in terms of overall budget expenditure and current and future trends in the healthcare and pharmaceutical budget. From 2016 to 2021, there was consistent growth of healthcare services and pharmaceutical spending: 82 and 80%, respectively. The largest expenditure was observed in a group containing chemotherapy medicines. The introduced budget cap is a relatively effective measure. Nevertheless, the high level of overspending and the pay-back amount requires better market environmental risk management.

Burzyńska and Pikala assess the mortality trends of senior Polish residents in the first two decades of the current century by the most frequent causes of death. The share of deaths due to diseases of the circulatory system decreased in all the subsamples. Among malignant neoplasms, lung and bronchial cancers accounted for the largest percentage of deaths. In this subgroup, the standardized death rates (SDRs) among males decreased, while those among females increased. In the 65–74 age group, the SDR value increased from 67.8 to 76.3, while in the 75-plus age group it increased from 112.1 to 155.2. As for influenza and pneumonia, the respective SDR demonstrated an upward trend. In recent years, rising trends in mortality driven by diseases of the digestive system were observed due to alcohol-induced liver disease for both genders in the 65–74 age group. In the 75-plus age group, falls were the most typical external cause of mortality.

Syria is the only country with an Arabic socialism legacy. Allaham et al. evaluate the perceived quality of healthcare services for two hospital types in Syria according to the five HEALTHQUAL dimensions. According to their results, service quality is better in private hospitals compared to public ones. Nevertheless, neither type of hospitals has exceptionally high scores in any of the examined HEALTHQUAL dimensions due to the crowded environment, the low wages of medical personnel, inadequate pricing policies, and, in general, the underdeveloped health insurance system.

Summing up, the articles in this Research Topic demonstrate that countries with a socialist legacy have a lot of similar problems related to the effective work of their respective healthcare systems. Moreover, implications are to a large extent generalized to a few other countries in this group and to some of the rapidly developing low- and middle-income countries of the Global South (8). Physicians, nurses, patients, and policymakers would benefit from further research on efficient healthcare delivery in the post-pandemic context, which is characterized by rapidly growing rates of digitalisation and high political instability.

## Author contributions

YT and MK prepared the manuscript draft, while MJ revised it for important intellectual content. All authors contributed to the article and approved the submitted version.
